# Macroscopic Hematuria as the Initial Presentation of Polycythemia Vera

**DOI:** 10.7759/cureus.10800

**Published:** 2020-10-05

**Authors:** Sebastião Carlos de Sousa Oliveira, Lucas Tadeu Rocha Santos, Mateus Aragão Esmeraldo, Osvaldo Pimentel de Oliveira Neto, Maycon Fellipe da Ponte

**Affiliations:** 1 Neurological Surgery, Federal University of Ceará, Sobral, BRA; 2 Internal Medicine, Federal University of Ceará, Sobral, BRA; 3 Neuroscience, Federal University of Ceará, Sobral, BRA

**Keywords:** hematuria, renal vein thrombosis, polycythemia vera

## Abstract

Polycythemia vera (PV) is a chronic myeloproliferative neoplasm (CMN) characterized by the accumulation of red blood cells, granulocytes and platelets in the peripheral blood. The main complications of PV are an increased risk of thrombosis, bleeding and transformation to myelodysplasia or acute leukemia. The authors report the case of a 28-year-old man with a complaint of macroscopic hematuria, low back pain and edema of the left arm associated with elevated hemoglobin, hematocrit and lactic dehydrogenase, leukocytosis and increased renal volume. Computed tomography of the chest and abdomen with contrast showed venous ectasia in the left upper limb and thrombosis of the right renal vein with extension to the inferior vena cava. A diagnosis of PV was confirmed by the presence of the JAK2 mutation and a bone marrow biopsy that showed panmyelosis. The patient was anticoagulated and treatment for PV was started with aspirin, phlebotomy and hydroxyurea. Then, the patient was discharged for outpatient follow-up with a hematologist. The case emphasizes the importance of clinical suspicion for atypical presentation of the disease in an unusual age range and of adequate etiological investigation of thrombosis in unusual sites.

## Introduction

In 1903, William Osler described polycythemia vera (PV), that belongs to a group of chronic myeloproliferative neoplasms (CMN), characterized by the expansion of terminal myeloid cells in the peripheral blood. Other examples of diseases of this group are essential thrombocythemia, myelofibrosis and chronic myeloid leukemia.

PV is distinguished from other CMN by the increase in red cells, granulocytes and platelets in the absence of a physiological stimulus. The clinical manifestations of the disease range from asymptomatic conditions with elevated hemoglobin or hematocrit to microvascular lesions that may generate symptoms such as headache, vertigo, visual disturbances or classical complications of the disease such as thrombosis or hemorrhage [1].

Such complications are more frequent in PV when compared to the rest of the CMN and they have effects on both patients' quality of life, morbidity and prognosis. Thrombosis in PV usually occurs before or during the diagnosis of the disease and in unusual sites such as splanchnic and cerebral veins [2].

More than 95% of PV cases have a Janus kinase 2 (JAK2) mutation, which replaces valine with phenylalanine (V167F), involving exons 14 or 12, as well as approximately 50% of patients with essential thrombocythemia and primary myelofibrosis [2]. JAK2 tyrosine is part of non-receptor tyrosine kinases and serves as analogous to the tyrosine kinase receptor of erythropoietin and thrombopoietin. Its constitutive activation may explain the formation of erythroid colonies independent of erythropoietin.

## Case presentation

A 28-year-old black man, with no previous comorbidities, presented to the emergency department with a complaint of low back pain, macroscopic hematuria and dysuria that started a week ago. Upon admission, the patient denied episodes of edema, changes in blood pressure levels, presence of foamy urine, pruritus or previous thrombosis. On physical examination, the patient had a blood pressure of 110 x 70 mmHg, a heart rate of 75 beats per minute, a respiratory rate of 17 breaths per minute, an axillary temperature of 36.8 ºC, oxygen saturation of 98% and no splenomegaly. Laboratory tests at admission showed hematuria without erythrocyte dysmorphism, elevated hemoglobin (17.6 g/dL) and hematocrit (59.3%), leukocytosis (14,670/mm³) and increased lactic dehydrogenase (415 IU/L).

**Figure 1:**
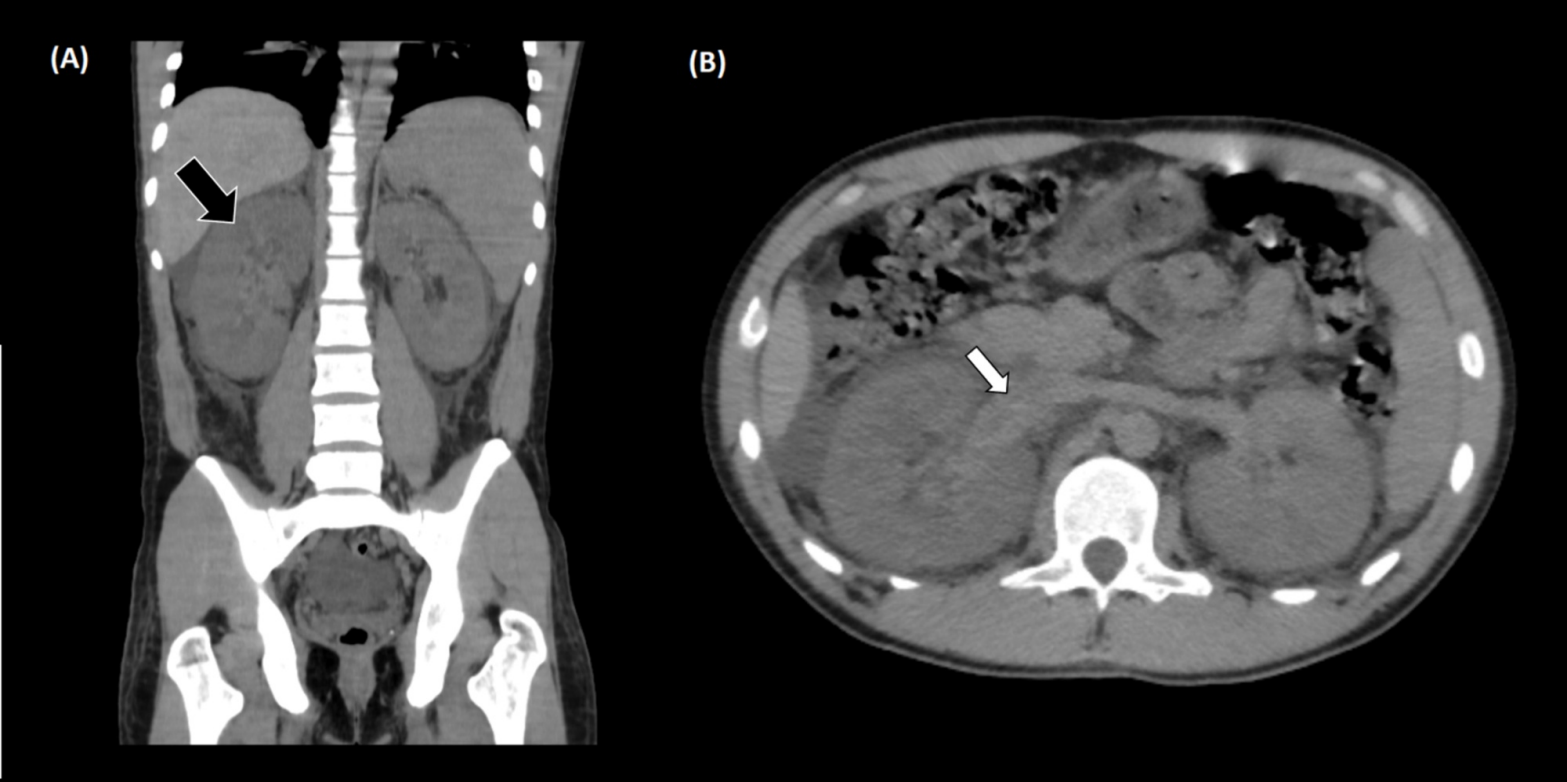
Non-contrast-enhanced CT of the abdomen Non-contrast-enhanced computed tomography of the abdomen showed an increase in the right kidney size (black arrow) and the presence of heterogeneous content in the right renal pelvis (white arrow), with no signs of obstruction due to urinary tract calculi.

**Figure 2:**
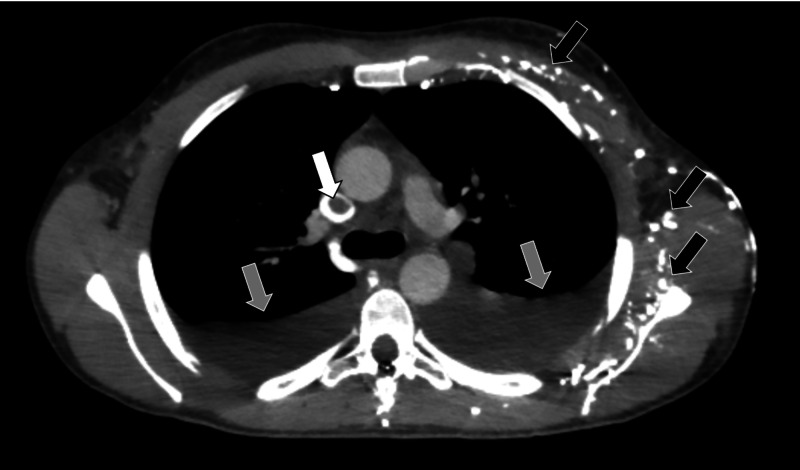
Contrast-enhanced CT of the chest Contrast-enhanced computed tomography of the chest showed suggestive signs of thrombus in the superior vena cava (white arrow), in addition to extensive venous ectasia in the left shoulder region (black arrows) and bilateral pleural effusion (gray arrows).

**Figure 3:**
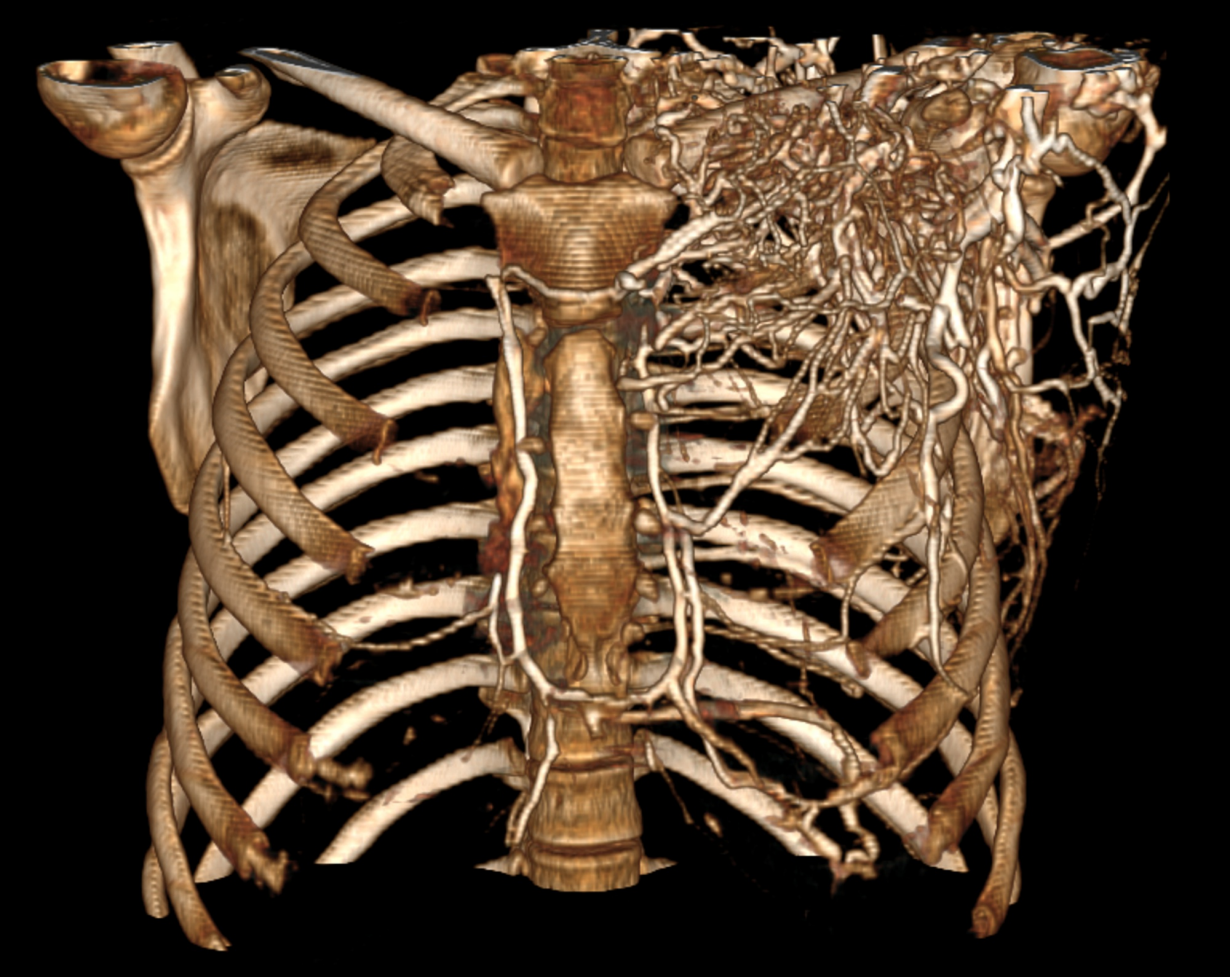
Three-dimensional reconstruction of the contrast-enhanced chest CT Three-dimensional reconstruction of the contrast-enhanced chest CT, showing venous ectasia and contrast enhancement in the venous system of the left upper limb.

**Figure 4:**
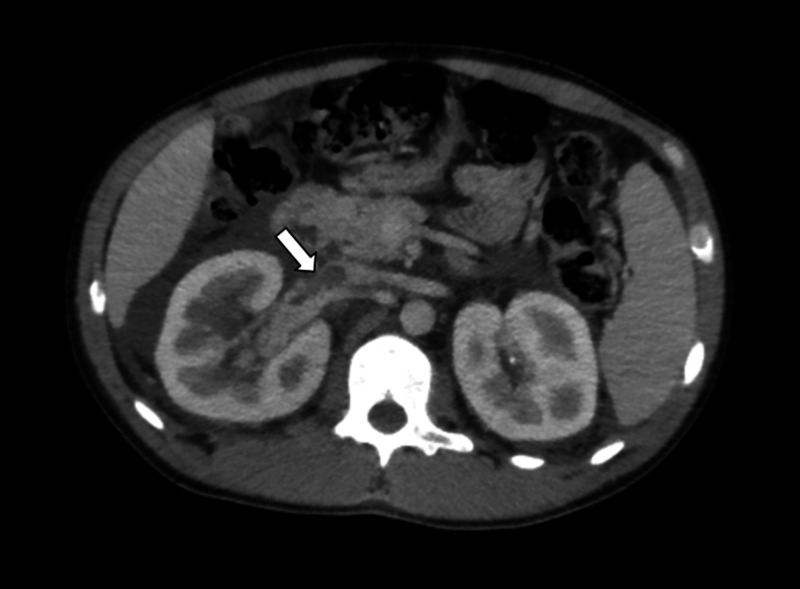
Contrast-enhanced CT of the abdomen Contrast-enhanced computed tomography of the abdomen in the portal venous phase showed a thrombus in the right renal vein with extension to the inferior vena cava (white arrow). Regarding the right kidney, it is possible to notice perirenal fluid and an increase in its volume.

A non-contrast-enhanced tomography of the abdomen was performed, which showed an increase in the right kidney size and the presence of heterogeneous content in the renal pelvis on the right, with no signs of obstruction due to urinary tract calculi (Figure 1). During hospitalization, the patient developed edema in the upper limbs and signs of venous congestion at the upper limbs. A contrast-enhanced computed tomography of the chest was performed, which showed suggestive signs of thrombus in the superior vena cava, in addition to extensive venous ectasia in the left shoulder region and to bilateral pleural effusion (Figures 2 and 3). A contrast-enhanced abdominal tomography showed signs of thrombosis in the right renal vein (Figure 4).

After finding extensive thrombosis, anticoagulation therapy was initiated according to the hospital protocol with enoxaparin and warfarin. Then, an investigation for coagulopathies was initiated, in addition to bone marrow biopsy that showed hypercellularity, mainly represented by erythrocyte, granulocytic and megakaryocytic series (Table 1).

**Table 1:** Etiological investigation of renal vein thrombosis GPL: IgG phospholipid units, MPL: IgM phospholipid units.

Examination	Patient	Reference range
Ferritin	107.4 ng/mL	23.9-336.2 ng/mL
Antithrombin III	114%	83-128%
Protein C	0.98 mg/L	0.7-1.6 mg/L
Protein S	57%	63.5-149%
Homocysteine	14 μmol/L	5.4-16.2 μmol/L
Anticardiolipin IgG	0.3 GPL	>20 GPL
Anticardiolipin IgM	3.3 MPL	>20 MPL
Lupus anticoagulant	1,1	<1.2
Factor V Leiden	Normal	Normal
Prothrombin gene mutation	Normal	Normal
Beta 2 microglobulin	1569 µg/mL	<2000 µg/mL
Somatic analysis of JAK 2	Presence of V167F mutation in exons 14 and 12	-
Bone marrow biopsy	Hypercellularity due to erythrocyte, granulocytic and megakaryocytic series	-

Associating the elevation of hemoglobin, hematocrit and lactic dehydrogenase, leukocytosis, hypercellular bone marrow biopsy and thrombosis in unusual sites, a diagnostic hypothesis of polycythemia vera was suggested and the detection of a JAK2 V167F mutation was requested, which resulted in a positive result. After establishing the diagnosis according to the criteria of the World Health Organization (WHO), the patient started therapy in the hematology service with phlebotomy, aspirin 100 mg/day and hydroxyurea 500 mg/day. The patient was discharged for specialized outpatient follow up.

## Discussion

The incidence of PV is 2.5 cases per 100.00 people and this disease affects any age group with an average age of 60 years. About 25% of cases occur before the sixtieth decade of life and only 10% before the age of 40 [3]. In the case described, the diagnosis of the disease is observed in a young individual, being a rare case in the medical literature. The literature lacks studies of how this population behaves when affected by the disease from the clinical and prognostic point of view.

The clinical picture of PV is attributed to the excess of inflammatory cytokines generated by the JAK 2 mutation, predisposing a systemic inflammatory state [1]. The most common clinical presentation of PV is the incidental finding of increased red blood cell count, high hemoglobin and hematocrit. Leukocytosis, thrombocytosis and splenomegaly are also early manifestations of the disease [3]. 

PV can present non-specific symptoms that hinder its diagnosis when analyzing cross-sectional studies involving the use of questionnaires. The most common complaint of patients with PV was fatigue, reported by 85% of patients, together with pruritus. These complaints were associated with a decrease in the quality of life of this population [4]. Another common complaint is aquagenic pruritus, described as pruritus after bathing with water, described in a questionnaire by 68% of patients with PV [5]. This symptom may be the initial clinical presentation of the disease and is described as a sensation of itching, tickling, stinging or burning associated or not with visible changes in the skin.

Due to the increase in blood viscosity and platelet reactivity, PV is characterized by signs and symptoms of microvascular involvement. The evaluation using the Myeloproliferative Neoplasm Symptom Assessment Form (MPN-SAF) identified the presence of the following vascular associated symptoms: lethargy (66%), depressed mood (65%), difficulty concentrating (61%), sexual dysfunction (57%), headache (52%) and dizziness (52%) [6]. The erythromelalgia, erythema, pallor or cyanosis associated with burning in hands and feet is a complication of microthrombotic disease, affecting approximately 29% of patients [3].

The physical examination of the patient with PV may present facial plethora, splenomegaly, hepatomegaly (fewer cases), systolic arterial hypertension (due to an increase in the mass of red cells), abrasions on the skin (suggestive of pruritus), stigmas of arterial or venous thrombotic events and gout tophi (due to the high turnover of hematopoietic cells) [3].

The case described does not present many symptoms described in the literature related to PV, increasing the challenge to establish the diagnosis in atypical cases, goal that was achieved mainly due to observation of thrombosis in unusual sites and laboratorial findings, which guided the diagnostic investigation of the case. 

Thrombosis and hemorrhage are the main complications of PV. These patients have a 3 to 13 times higher risk of the cerebrovascular event, myocardial infarction, superficial thrombophlebitis, thrombosis and pulmonary embolism when compared with matched controls regarding sex and age. In about 34% of cases, thrombosis occurs before or during diagnosis, and in 55% of cases, it occurs in unusual sites such as splanchnic and brain veins. Arterial thrombosis is more frequent (16-27%) compared to venous thrombosis (7-12%) and both represent the main cause of morbidity and mortality in patients with PV, directly influencing the treatment chosen for the patient [2].

The main risk factors for thrombosis in PV are age over 65 years and a history of thrombosis [2]. In a study performed by the European Collaboration on Low-Dose Aspirin in Polycythemia Vera (ECLAP), age >65 years (relative risk 2.08 [95% CI, 1.25-3.45]) and history of the thrombotic event (relative risk 2.09 [95% CI, 1.55-2.81]) were the two prognostic indicators of the cardiovascular event. Sex also influences the risk for thrombosis, being the arterial events most common in men (18% versus 14%) and the venous events most common in women (9.3% versus 5.4%) [7]. The elevated hematocrit increases blood viscosity and acts as a risk factor for thrombosis, being indicated the performance of phlebotomy in PV. Other risk factors are studied, such as hypertension in low-risk patients, leukocytosis and elevated C-reactive protein [8].

In the case described, the initial presentation of PV is macroscopic hematuria resulting from thrombosis of the right renal vein with extension to the inferior vena cava. The clinical picture of renal vein thrombosis is usually described as low back pain due to renal infarction, macroscopic or microscopic hematuria, increased lactic dehydrogenase and increased kidney size [9]. Renal vein thrombosis is a rare disease that can occur as a consequence of nephrotic syndrome, especially in membranous nephropathy with proteinuria >10 g/day and serum albumin <2 g/dL. Hipovolemia in newborns and hereditary thrombophilia are other common causes. Thus, the authors present a case of PV with an unusual presentation and site of thrombosis in a young patient. 

The study by the International Working Group for Myeloproliferative Neoplasms Research and Treatment (IWG-MRT) summarizes the following laboratory changes in 1545 patients with PV: elevated hemoglobin (median 18.4 g/dL [15.1-26.5]), elevated hematocrit (55% [36-78%]), thrombocytosis in 53% of patients, 50% increase in lactic dehydrogenase and the presence of the JAK 2 mutation in 98% [3]. Such findings were compatible with the laboratory alterations described in the case mentioned previously. The diagnostic criteria for WHO PV are as follows (Table 2).

**Table 2:** WHO diagnostic criteria for polycythemia vera

Major criteria
1. Hemoglobin > 16.5 g/dL in men or >16 g/dL in women or hematocrit >49% in men or >48% in women
2. Bone marrow biopsy with hypercellularity and panmyelosis
3. Presence of JAK2 V167F or exon 12 mutation
Minor criteria
1. Low erythropoietin level

**Figure 5:**
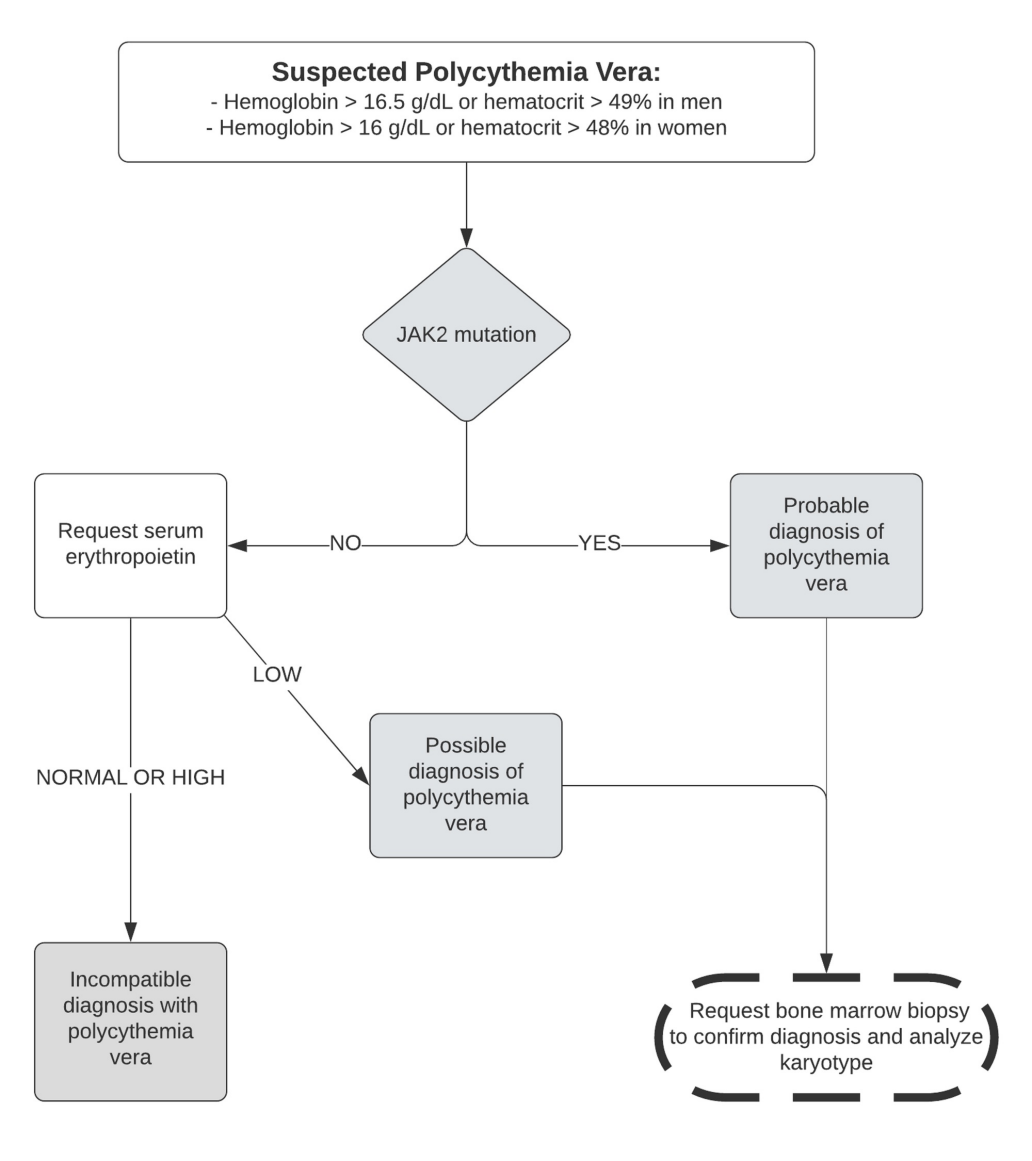
Practical algorithm for suspected cases of polycythemia vera

The diagnosis of PV requires the three major criteria or the first two major criteria and the minor criterion [10]. The flowchart (Figure 5) can be helpful in the investigation of PV. In the case described, the investigation started after analysis of elevated hemoglobin and hematocrit associated with renal vein thrombosis extending to the lower vena cava and venous ectasia of the left upper limb. In addition, an investigation was carried out for hereditary thrombophilia. After the result of the JAK2 mutation and bone marrow biopsy, the diagnosis of PV was confirmed by WHO criteria.

The goals of the treatment of PV consist in the control of symptoms (itching, erythromelalgia) and the prevention of complications such as thrombosis or hematological transformation (myelofibrosis, acute myeloid leukemia and myelodysplastic syndromes). To define the appropriate treatment, patients are stratified by the risk of thrombotic episodes, being considered high-risk patients the ones with age over 60 years or a history of thrombosis and low-risk those younger than 60 years and without a history of thrombosis.

The standard treatment, regardless of risk, consists of low-dose aspirin (40 to 100 mg, once or twice daily) and phlebotomy with a target hematocrit of less than 45% for all patients or 45% for men and 42% for according to expert suggestion. Aspirin offers a 60% reduction in the risk of myocardial infarction, stroke, pulmonary embolism and death from cardiovascular causes (relative risk 0.4; 95% CI, 0.19-0.91; p = 0.03) without increasing the risk of major bleeding [11]. Phlebotomy with a more intensive target (<45%), when compared to less intensive treatment (hematocrit between 45% and 50%), shows a significant reduction in the risk of cardiovascular death and thrombosis (hazard ratio 3.91; p = 0.07) [11]. Phlebotomy has no difference in progression to primary myelofibrosis, myelodysplasia, acute leukemia and bleeding.

In low-risk patients, the treatment of PV includes the assessment and management of risk factors for cardiovascular diseases such as blood pressure control, smoking cessation, weight reduction and physical activity. In high-risk patients, the main difference is the use of cytoreductive therapy with hydroxyurea, interferon-α, busulfan or ruxolitinib. The use of cytoreductive agents in low-risk patients is restricted to cases with symptoms refractory to standard treatment, progressive increase in leukocytes or platelets, symptomatic or progressive splenomegaly or patients who cannot tolerate phlebotomy [12].

Hydroxyurea is the first-line therapy to obtain cytoreduction in PV, being used in the initial dose of 15-20 mg/kg/day, with dose adjustments to achieve platelet count between 100.000 and 400.000/µL. Approximately 10% of patients present treatment failure with hydroxyurea. In these groups, other cytoreductive agents become therapeutic options. Interferon-α can be used in patients with refractory symptoms to hydroxyurea or may be used as first-line treatment in patients younger than 40 years and that have a desire for offspring. The busulfan is used in older patients (>60 years) that failed to obtain myelosuppression with other agents. In the failure of the aforementioned agents, one option is the use of ruxolitinib, an inhibitor of the Janus kinase complex [10].

**Figure 6:**
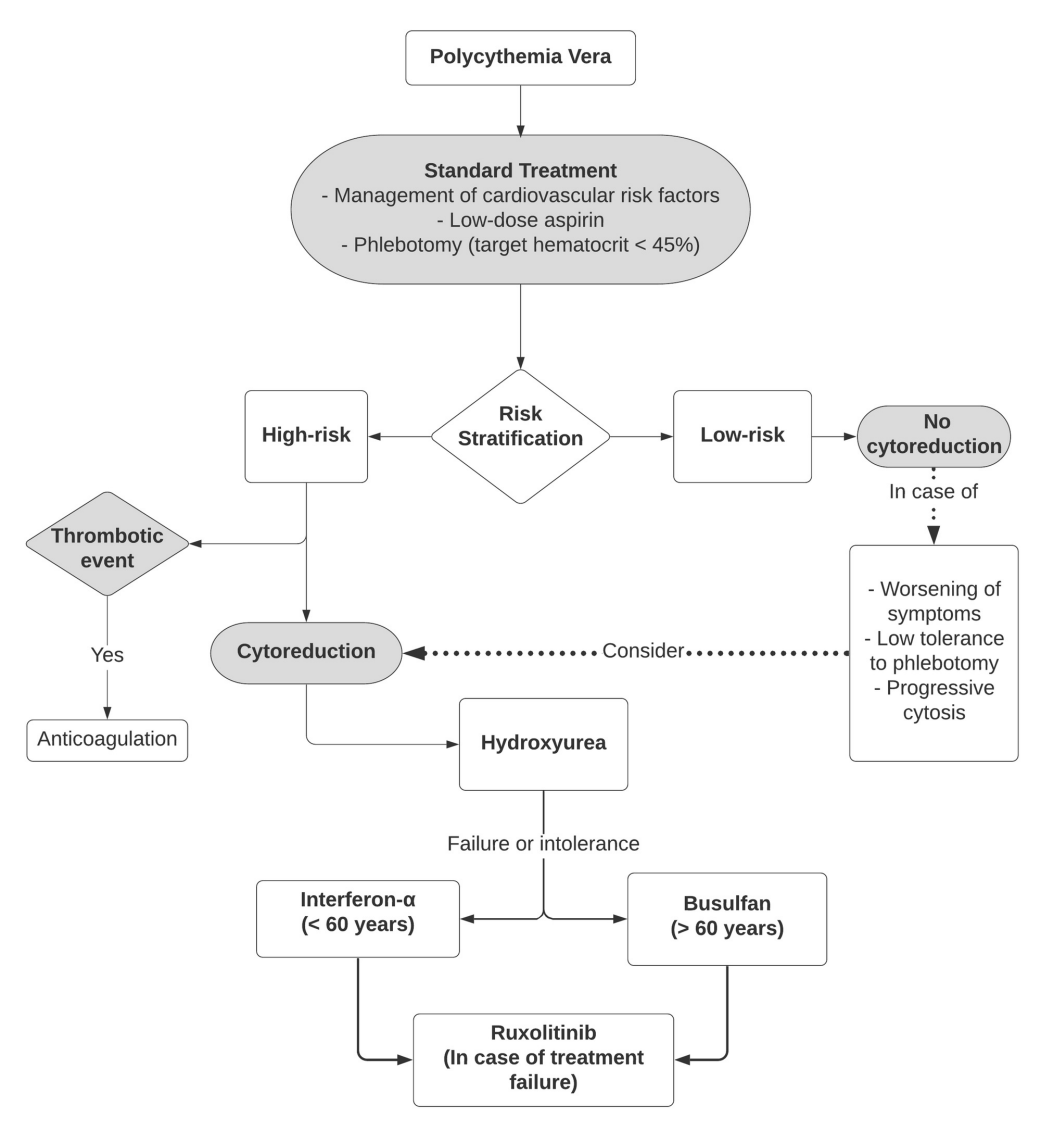
Algorithm for current therapeutic options for polycythemia vera

Patients with thrombotic episodes should be anticoagulated in accordance with routine protocols and those with bleeding should have aspirin and anticoagulation suspended and perform research for von Willebrand disease. In the case described, standard therapy with aspirin and phlebotomy was instituted, as well as cytoreductive therapy with hydroxyurea, since the patient was in the high-risk group, and anticoagulation was prescribed due to the thrombotic episodes. The flowchart (Figure 6) presents the current therapeutic options for PV.

## Conclusions

The present case describes the clinical picture of a young patient with macroscopic hematuria, elevated hematocrit and red blood cell count, renal thrombosis with extension to the inferior vena cava and venous ectasia in the left upper limb caused by PV.

The case emphasizes the importance of clinical suspicion for atypical presentation of the disease, in an unusual age range, and the adequate etiological investigation of thrombosis in unusual sites.
